# Trends and Practices in Bariatric Surgery in Egypt: Insights on Esophagogastroduodenoscopy (EGD) Utilization and Surgical Volumes

**DOI:** 10.1007/s11695-025-07846-0

**Published:** 2025-05-20

**Authors:** Mohamed H. Zidan, Ahmed Abokhozima, Mohannad I. A. Gaber, Ahmed Amgad, Hashem Altabbaa, Hassan El-Masry, Mohammed Alokl, Reda Fawzy Ali, Ahmed Abo Elmagd, Aliaa Selim, Khaled Gawdat, Collaborative Egyptian National Survey Group, Abdelrahman Mohamed Salah, Ahmad Ali maklad, Ahmed Amin, Ahmed El Assal, Ahmed Fadaly Hussein, Ahmed Gamal Abdallah, Ahmed H. Hussein, Ahmed H. Darwish, Ahmed Mehrez Gad, Ahmed Mohammed Farid Mahmoud Hamdy Mansour, Ahmed Saad Khalil, Ahmed Taher Mohamed Yousef, Ahmed Yousry, Aiman Ismaeil, Alaa Abbass S. Moustafa, Alaa Abdelaty Mahmoud, Alaa Badawy, Alaa Sewefy, Anwar Ashraf Abouelnasr, Ashraf Ahmed Elattar, Ayman Kamal, Emad Abdallah, Faheem Aly Elbassiony, Hany Maurice Sabri Mikhail, Hassan Z. Shaker, Heba Elkomy, Hesham Abdallah, Hosam Hamed, Hosam Mohamed Elghadban, Hossam Ramadan Moussa, Ibrahim Karam Elsehwagy, Ibrahim Shalaby, Islam Abdelkhalek, Kareem Farouk, Karim Sabry, Khaled Katri, Mahmoud Saad Saad, Mario Saba, Mohamed Abd Allah, Mohamed Abdelaziz, Mohamed Abouzeid, Mohamed Elemawy, Mohamed Elsaied Aboelnadar Abdelaty, Mohamed Fikry, Mohamed Hashish, Mohamed Ibrahim, Mohamed Khalaf, Mohamed Maher El Araby, Mohamed Mahmoud Abdalgaliel, Mohamed Moharem Okba, Mohamed Mokhtar Arafat, Mohamed Mosaad Kandel, Mohamed Mourad, Mohamed Nasr Shazly, Mohamed Sharshar, Mohammad Hamdy Abo-Ryia, Mohammed Ammar, Mohammed Hany, Mohammed Mustafa Mohammed, Mossad Anwar Hemida, Mostafa Gamal, Mostafa Refaie Elkeleny, Omar Rady Khairat Hammad, Ragab Mohammed Seddik, Ramy Helmy, Salah El Sakhawy, Sherif Mohamed Zeidan, Tamer Abdelbaki, Tamer Elmahdy, Tamer Mohamed Nabil Sayed, Tarek Abouzeid Osman, Wael Nabil, Waleed Allam, Yasser Amer

**Affiliations:** 1 Alexandria University, Alexandria, Egypt; 2 Alexandria Main University Hospital, Alexandria University, Alexandria, Egypt; 3 El-Ekbal Hospital, Alexandria, Egypt; 4 Kingston Hospital, NHS Foundation Trust, Kingston, England UK; 5 Helwan University, Cairo, Egypt; 6 Faculty of Medicine, Helwan University, Cairo, Egypt; 7 The Research Papyrus Lab, Alexandria, Egypt; 8 Kafr Elsheikh University Hospital, Kafr Elsheikh, Egypt

**Keywords:** Esophagogastroduodenoscopy, Bariatric surgery, Egypt, Economic constraints, IFSO guidelines

## Abstract

**Background:**

Esophagogastroduodenoscopy (EGD) is crucial in bariatric surgery for detecting gastro-esophageal conditions and incidental pathologies, impacting surgical decisions and outcomes. The International Federation for the Surgery of Obesity and Metabolic Disorders (IFSO) recommends routine EGD before and after bariatric procedures to identify incidental pathologies. However, global adherence to these guidelines varies, especially in resource-constrained settings where economic limitations often dictate practice patterns. This study adapts a survey by Quake et al. (2022) to the Egyptian context, offering a comprehensive analysis of EGD utilization alongside broader trends in metabolic and bariatric surgery (MBS) practices in Egypt.

**Methods:**

A survey adapted from Quake et al. (2022) was tailored to assess trends in metabolic and bariatric surgery (MBS) practices in Egypt. Conducted between April and August 2024 with a response rate of 53.3%, the survey targeted Egyptian bariatric surgeons. It evaluated EGD utilization, surgical expertise, institutional volumes, types of procedures, revisional surgeries, and adherence to the 2020 IFSO position statement. Data was collected through Google Forms and analyzed for trends, challenges, and gaps in practice, focusing on economic constraints and guideline implementation.

**Results:**

Among the 80 respondents, 88.8% were consultants, with 73.8% performing over 100 surgeries annually. The volume of bariatric procedures increased from 2021 to 2023, with significant growth in sleeve gastrectomy (SG) and single-anastomosis sleeve ileal (SASI) bypasses/bipartition. Revisional surgeries were most commonly Roux-en-Y gastric bypass (RYGB). Despite this growth, EGD utilization remained limited. Pre-operatively, only 12.5% of surgeons performed EGD routinely for all patients, while 67.5% used it selectively based on patient or procedural factors. Post-operative EGD at one year was routinely offered by just 3.8% of surgeons, with 55% not routinely using it at all. Institutional and economic factors influenced these practices; surgeons in high-volume or private settings were more likely to adopt selective EGD use. Awareness of the 2020 IFSO guidelines showed a minimal impact on EGD practices, suggesting that financial considerations often outweigh clinical recommendations.

**Conclusions:**

This study highlights critical trends in bariatric surgery practices in Egypt, including increasing procedural volumes and the limited utilization of EGD. Economic constraints remain the predominant barrier to routine EGD use, despite its potential to improve surgical outcomes by identifying incidental pathologies. Enhancing patient care requires establishing a national registry, upgrading training programs, and implementing observerships to align with international standards are pivotal in advancing bariatric care in Egypt and guaranteeing high-caliber, evidence-based patient care.

**Supplementary Information:**

The online version contains supplementary material available at 10.1007/s11695-025-07846-0.

## Introduction

The use of esophagogastroduodenoscopy (EGD) in bariatric surgery has been widely acknowledged for its ability to detect various gastro-esophageal diseases and incidental pathologies [1–4]. The results can range from hiatal hernias, gastroesophageal reflux disease, and Barrett’s esophagus, to various incidental pathologies such as gastric gastrointestinal stromal tumors (GISTs), gastric polyps, and gastric malignancies. These findings can have a substantial impact on the surgical approach, ultimately improving postoperative results [1].

In 2020, the International Federation for the Surgery of Obesity and Metabolic Disorders (IFSO) recommended the routine use of EGD before and after bariatric surgery [5]. They emphasized the favorable use of EGD before bariatric procedures, particularly in patients with upper gastrointestinal symptoms, and before gastric bypass procedures, such as one-anastomosis gastric bypass (OAGB) and Roux-en-Y gastric bypass (RYGB), where part of the stomach will be inaccessible.

The IFSO statement also stressed the necessity of EGD after bariatric surgery at 1 year, and then every 2–3 years for patients who have undergone sleeve gastrectomies (SG) or OAGB, to facilitate early detection of Barrett’s esophagus or upper GI malignancies. Additionally, EGD should be initiated after any bariatric surgery based on post-operative upper GI symptoms.

Despite the IFSO’s ongoing efforts to standardize the approach to bariatric surgeries, various shortcomings in different healthcare systems globally may impact these efforts. This is because the recommendations were based primarily on the advantages of EGD, while disregarding the economic burden of such investigations, particularly in developing countries.

In 2022, Quake et al. conducted an international survey to assess the use of EGD in bariatric surgeries [6]. The survey aimed to explore the factors influencing EGD’s selective or routine use, surgeons’ awareness of IFSO guidelines, and the potential impact of institutional bariatric surgery volume on EGD practices. It also examined the frequency of EGD surveillance after specific procedures like SG and OAGB, and the challenges and considerations surrounding long-term follow-up endoscopy.

This study aims to enhance our understanding of the trends in bariatric and metabolic surgery (MBS) and the use of esophagogastroduodenoscopy (EGD) in Egypt. By adapting the survey developed by Quake et al. for the Egyptian context, it assesses the practices of surgeons, the volume of surgeries performed, and adherence to established guidelines, seeking to identify actionable solutions for improving the quality of care and aligning local practices with international standards.

While this study is based in Egypt, the study aims to highlight challenges that may apply to other resource-limited settings where economic factors influence adherence to international guidelines on EGD utilization in bariatric surgery. Studies from other low- and middle-income countries have also shown disparities in EGD utilization due to financial constraints, with varying adherence to international recommendations [3, 6–10]. Highlighting Egypt’s experience can provide insights into how financial constraints impact surgical practice both in developing countries and worldwide, highlighting the necessity of tailored guidelines for these settings.

## Methods

### Conceptualization and Data Collection

The survey used in this research was based on a previously designed international survey by Quake et al. [6] and was adapted for the Egyptian context. It was aimed at active Egyptian bariatric surgeons, including those associated with the Egyptian Society of Bariatric Surgeons (ESBS) or current IFSO 2024 members. All collaborative surgeons are actively practicing bariatric surgeries in either private or governmental institutes in Egypt.

Contacts were collected through different WhatsApp, and social media groups, that kept the contact of the participants visible. Furthermore, some surgeons were reached through local institutes and private hospitals’ contact details. Additionally, all current members of the IFSO members were checked from the online platform at https://www.ifso.com/find-a-member/country-lt-64/, and contacts were retrieved via social media platforms.

Collectively, we reached out to 150 contacts, including the 34 current IFSO members (2024) (Table 1). Google Forms was used to conduct the survey, using the same questionnaire as Quake et al. All participants were asked to fill out a consent form, and agree that their names, and affiliations would be used for research conduction.

**Table 1:** Expertise, memberships, and type of affiliation of Participating MBS surgeons

Expertise, memberships, and type of affiliation of Participating MBS surgeons	*N* (%)
**IFSO member**	52 (65)
**Current 2024 IFSO member**	15 (18.8)
**ESBS member**	58 (72.5)
**Grade/ level**	
- Consultant	71 (88.8)
- Specialist	9 (11.3)
- Residents	0 (0)
**The type of affiliation/unit in which you practice bariatric surgery**	
Private Institution	25 (31.3)
Governmental Institution	5 (6.3)
Both Private and Governmental institutions	50 (62.5)

All Invitations were initially sent in April 2024 via WhatsApp messages, or emails, followed by reminders in June and July 2024, and the survey responses were closed on the 20th of August 2024. We received 80 responses, resulting in a response rate of 53.3%. This included feedback only from 15 out of the 34 current IFSO members (44.1% response rate). All participants agreed to participate in this study (Table 2).

**Table 2:** List of Collaborators and Their Affiliated Cities, full collaborative authors’ details are available under *“Collaborative Egyptian National Survey Group”* authorship

Name	City
Abdelrahman Mohamed Salah	Minya
Ahmad Ali maklad	Ismailia
Ahmed Amin	El Alamein
Ahmed El Assal	Alexandria
Ahmed Fadaly Hussein	El Mansoura
Ahmed Gamal Abdallah	Cairo
Ahmed H. Hussein	Ismailia
Ahmed H.Darwish	Alexandria
Ahmed Mehrez Gad	Gharbia
Ahmed Mohammed Farid Mahmoud Hamdy Mansour	Alexandria
Ahmed Saad Khalil	Alexandria
Ahmed Taher Mohamed Yousef	Alexandria
Ahmed Yousry	Cairo
Aiman Ismaeil	Aswan
Alaa Abbass S. Moustafa	Cairo
Alaa Abdelaty Mahmoud	Alexandria
Alaa Badawy	Alexandria
Alaa Sewefy	Minya
Anwar Ashraf Abouelnasr	Alexandria
Ashraf Ahmed Elattar	Tanta
Ayman Kamal	Cairo
Emad Abdallah	El Mansoura
Faheem Aly Elbassiony	Cairo
Hany Maurice Sabri mikhail	Cairo
Hassan Z Shaker	Cairo
Heba Elkomy	Alexandria
Hesham Abdallah	El Mansoura
Hosam Hamed	El Mansoura
Hosam Mohamed Elghadban	El Mansoura
Hossam Ramadan Moussa	Tanta
Ibrahim Karam Elsehwagy	Alexandria
Ibrahim Shalaby	Alexandria
Islam Abdelkhalek	Alexandria
Kareem Farouk	Tanta
Karim Sabry	Cairo
Khaled Gawdat	Cairo
Khaled Katri	Alexandria
Mahmoud Saad Saad	Alexandria
Mario Saba	Giza
Mohamed Abd Allah	El Mansoura
Mohamed Abdelaziz	Minya
Mohamed Abouzeid	Cairo
Mohamed Elemawy	Kafr Elsheikh
Mohamed Elsaied Aboelnadar Abdelaty	Beheira
Mohamed Fikry	El Mansoura
Mohamed Hashish	Tanta
Mohamed Ibrahim	Alexandria
Mohamed Khalaf	Minya
Mohamed Maher El Araby	Cairo
Mohamed Mahmoud Abdalgaliel	Beheira
Mohamed Moharem Okba	Alexandria
Mohamed Mokhtar Arafat	Alexandria
Mohamed Mosaad Kandel	Port Said
Mohamed Mourad	Alexandria
Mohamed Nasr Shazly	Cairo
Mohamed Sharshar	Alexandria
Mohammad Hamdy Abo-ryia	Tanta
Mohammed Ammar	Alexandria
Mohammed Atef Alokl	Alexandria
Mohammed Hany	Alexandria
Mohammed Mustafa Mohammed	Zagazig
Mossad Anwar Hemida	Beheira
Mostafa Gamal	Alexandria
Mostafa Refaie Elkeleny	Alexandria
Omar Rady Khairat Hammad	El Mansoura
Ragab Mohammed Seddik	Alexandria
Ramy Helmy	Cairo
Salah El Sakhawy	Alexandria
Sherif Mohamed Zeidan	Alexandria
Tamer Abdelbaki	Alexandria
Tamer Elmahdy	Tanta
Tamer Mohamed Nabil Sayed	Cairo
Tarek Abouzeid Osman	Cairo
Wael Nabil	Alexandria
Waleed Allam	Beheira
Yasser Amer	Cairo

### Assessment of Surgical Expertise and Surgical Caseload

To assess the proficiency and caseload of bariatric surgeons participating in the study, we categorized surgeons as specialists or consultants. Moreover, for a surgeon to be considered an expert in the field, they needed to have carried out a minimum of 100 bariatric surgeries within a single year. The survey comprised specific inquiries aimed at determining the volume of surgeries performed by each surgeon over the preceding 3 years, enabling us to identify those who met or surpassed this threshold.

### Institutional Volumes and Geographic Mapping

**Fig. 1:**
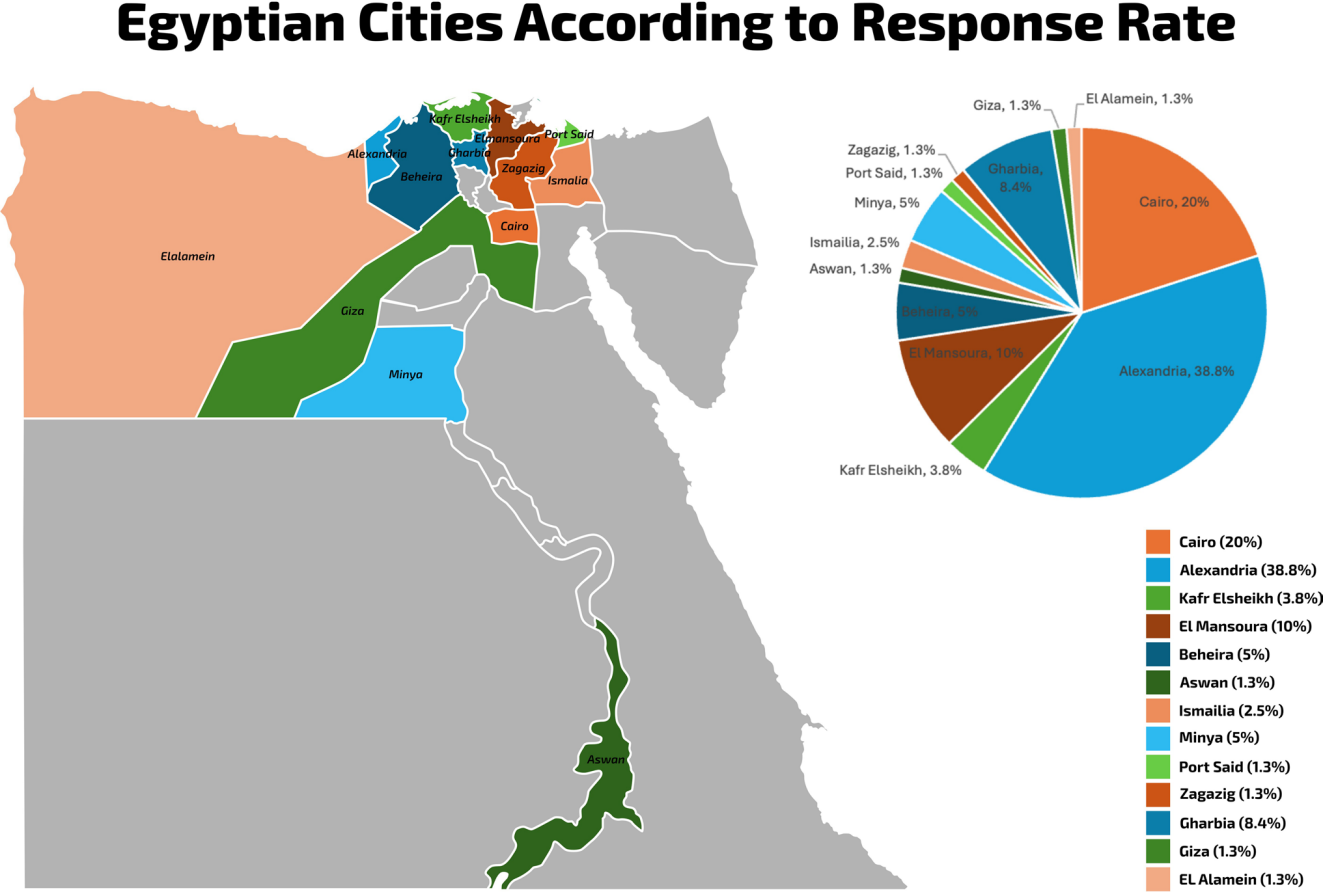
A geographical mapping of Egypt showing the response rates of the respondents according to their cities

The survey gathered information on the primary affiliations of participants and the types of hospitals where the respondents conduct metabolic and bariatric surgeries (MBS) procedures, including governmental hospitals, private hospitals, or both (Table 1). Each respondent was asked about the volume of bariatric procedures performed at their hospital, additionally, the respondents were enquired about the caseload performed as a primary surgeon (Table 3). To avoid duplicating results, responses were grouped by hospital and affiliation. The centers were then classified as High-Volume or Low-Volume based on these groupings. Furthermore, geographic estimates of respondents and their practices were provided by identifying their city of practice and conducting geographical mapping of these locations in Egypt (Fig. 1).

**Table 3:** Annual volume of MBS Performed by the surgeons and their centers in years 2021, 2022, and 2023

Estimated ranges of the volume of MBS procedures involved in each year	The volume of MBS the respondent is involved in each year as a primary surgeon *N* (%)	The volume of MBS undertaken at the surgeon’s center *N* (%)
**Year 2021**		
< 25 cases	21 (26.3)	12 (15)
25–49 cases	10 (12.5)	7 (8.8)
50–100 cases	12 (15)	15 (18.8)
> 100 cases	37 (46.3)	46 (57.5)
**Year 2022**		
< 25 cases	15 (18.8)	7 (8.8)
25–49 cases	12 (15)	11 (13.8)
50–100 cases	11 (13.8)	7 (8.8)
> 100 cases	42 (52.5)	55 (68.8)
**Year 2023**		
< 25 cases	14 (17.5)	5 (6.3)
25–49 cases	10 (12.5)	10 (12.5)
50–100 cases	10 (12.5)	6 (7.5)
> 100 cases	46 (57.5)	59 (73.8)

### Types of Primary Bariatric Procedures Performed and the Number of Surgeons Performed/Performing the Surgery

In the survey, we encompassed all approved primary metabolic and bariatric surgeries (MBS), including sleeve gastrectomies (SG), one-anastomosis gastric bypass (OAGB), Roux-en-Y gastric bypass (RYGB), banded sleeve gastrectomy (BSG), banded Roux-en-Y gastric bypass (BRYGB), banded one-anastomosis gastric bypass (BOAGB), single-anastomosis duodeno–ileal bypass with sleeve gastrectomy (SADI-S), and adjustable gastric band (AGB). We then requested the surgeons to indicate the volume range of these surgeries they performed in 2021, 2022, and 2023—categorized as < 25 cases, 25–49 cases, 50–100 cases, > 100 cases, or none (Table 4).

**Table 4:** Types and Range of MBS Performed Annually in years 2021, 2022, and 2023, with the maximum number of surgeons performing the procedure over the years

Type and range of performed surgeries		Year 2021 *N* (%)	Year 2022 *N* (%)	Year 2023 *N* (%)	Maximum number *N* (%) of Surgeons performing the procedure over the years
**Primary SG**					
< 25 cases		22 (27.5)	16 (20)	18 (22.5)	80 (100)
25–49 cases		11 (13.8)	13 (16.3)	15 (18.8)	
50–100 cases		15 (18.8)	16 (20)	15 (18.8)	
> 100 cases		27 (33.8)	30 (37.5)	32 (40)	
None		5 (6.3)	5 (6.3)	0 (0)	
Surgeons performing the surgery		75 (93.8)	75 (93.8)	80 (100)	
**Primary banded SG**					
< 25 cases		10 (12.5)	11 (13.8)	12 (15)	17 (21.3)
25–49 cases		1 (1.3)	1 (1.3)	2 (2.5)	
50–100 cases		5 (6.3)	2 (2.5)	1 (1.3)	
> 100 cases		1 (1.3)	1 (1.3)	2 (2.5)	
None		63 (78.8)	65 (81.3)	63 (78.8)	
Surgeons performing the surgery		17 (21.3)	15 (18.6)	17 (21.3)	
**Primary OAGB**					
< 25 cases		33 (41.3)	29 (36.3)	27 (33.8)	65 (81.3)
25–49 cases		6 (7.5)	12 (15)	13 (16.3)	
50–100 cases		13 (16.3)	11 (13.8)	9 (11.3)	
> 100 cases		12 (15)	13 (16.3)	14 (17.5)	
None		16 (20)	15 (18.8)	17 (21.3)	
Surgeons performing the surgery		64 (80)	65 (81.3)	63 (78.8)	
**Primary banded OAGB**					
< 25 cases		3 (3.8)	4 (5)	7 (8.8)	8 (10)
25–49 cases		1 (1.3)	0 (0)	0 (0)	
50–100 cases		3 (3.8)	1 (1.3)	1 (1.3)	
> 100 cases		0 (0)	0 (0)	0 (0)	
None		73 (91.3)	75 (93.8)	72 (90)	
Surgeons performing the surgery		7 (8.8)	5 (6.3)	8 (10)	
**Primary RYGB**					
< 25 cases		36 (45)	31 (38.8)	34 (42.5)	64 (80)
25–49 cases		9 (11.3)	14 (17.5)	17 (21.3)	
50–100 cases		7 (8.8)	6 (7.5)	6 (7.5)	
> 100 cases		7 (8.8)	6 (7.5)	7 (8.8)	
None		21 (26.3)	22 (28.7)	16 (20)	
Surgeons performing the surgery		59 (73.8)	58 (72.5)	64 (80)	
**Primary banded RYGB**					
< 25 cases		8 (10)	8 (10)	8 (10)	11 (13.8)
25–49 cases		0 (0)	0 (0)	0 (0)	
50–100 cases		2 (2.5)	0 (0)	0 (0)	
> 100 cases		1 (1.3)	1 (1.3)	2 (2.5)	
None		69 (86.3)	71 (88.8)	70 (87.5)	
Surgeons performing the surgery		11 (13.8)	9 (11.3)	10 (12.5)	
**Primary SADI-S**					
< 25 cases		15 (18.8)	13 (16.3)	12 (15)	17 (21.3)
25–49 cases		0 (0)	0 (0)	2 (2.5)	
50-100 cases		2 (2.5)	1 (1.3)	1 (1.3)	
> 100 cases		0 (0)	0 (0)	0 (0)	
None		63 (78.8)	66 (82.5)	65 (81.3)	
Surgeons performing the surgery		17 (21.3)	14 (17.5)	15 (18.8)	
**Primary AGB**					
< 25 cases		3 (3.8)	3 (3.8)	6 (7.5)	6 (7.5)
25–49 cases		0 (0)	0 (0)	0 (0)	
50–100 cases		1 (1.3)	0 (0)	0 (0)	
> 100 cases		0 (0)	0 (0)	0 (0)	
None		76 (95)	77 (96.3)	74 (92.5)	
Surgeons performing the surgery		4 (5)	3 (3.8)	6 (7.5)	
**Other Primary MBS**					
< 25 cases		24 (30)	27 (33.8)	21 (26.3)	38 (47.5)
25–49 cases		5 (6.3)	2 (2.5)	5 (6.3)	
50–100 cases		3 (3.8)	3 (3.8)	4 (5)	
> 100 cases		3 (3.8)	6 (7.5)	7 (8.8)	
None		45 (56.3)	42 (52.5)	42 (52.5)	
Surgeons performing the surgery		35 (43.8)	38 (47.5)	38 (47.5)	
**Most common Other Primary MBS**					
SASI		25 (31.3)	26 (32.5)	28 (35)	28 (35)
SASJ		11 (13.8)	11 (13.8)	11 (13.8)	11 (13.8)
Plication		3 (3.8)	3 (3.8)	4 (5)	4 (5)
Re-sectional OAGB		0 (0)	0 (0)	1 (1.3)	1 (1.3)
SAPI		0 (0)	0 (0)	1 (1.3)	1 (1.3)
**Revisional Surgeries**					
< 25 cases		27 (33.8)	27 (33.8)	30 (37.5)	67 (83.8)
25–49 cases		13 (16.3)	20 (25)	14 (17.5)	
50–100 cases		16 (20)	9 (11.3)	9 (11.3)	
> 100 cases		10 (12.5)	10 (12.5)	11 (13.8)	
None		14 (17.5)	13 (16.3)	14 (17.5)	
Surgeons performing Revisional surgeries		66 (82.5)	67 (83.8)	66 (82.5)	
**Most common Revisional intervention offered to the patient**					
RYGB		47 (58.8)	46 (57.5)	46 (57.5)	47 (58.8)
OAGB		19 (23.8)	18 (22.5)	16 (20)	19 (23.8)
SASJ		1 (1.3)	1 (1.3)	1 (1.3)	1 (1.3)
SASI		1 (1.3)	4 (5)	4 (5)	4 (5)
LSG/ Re-Sleeve		2 (2.5)	3 (3.8)	1 (1.3)	3 (3.8)
Banded Re-sleeve		1 (1.3)	1 (1.3)	0 (0)	1 (1.3)
Banded RYGB		1 (1.3)	1 (1.3)	1 (1.3)	1 (1.3)

We also enquired about the performance of other primary MBSs and the scope and variety of these procedures. This encompassed the volume of procedures performed and the specific types of procedures, with the option for multiple responses (Table 4).

Since the data was structured quantitatively, which posed challenges in assessing the trend of the surgeries performed, we computed the trend of the surgeries performed by the number of surgeons performing each MBS. Consequently, we derived the trend of the performing surgeons for each procedure, rather than the volume of the surgeries performed. However, the responses provide a rough estimate of the volume of MBS performed.

### Revisional Surgeries: Volume, Types, and Correlation to the Surgeon’s Expertise

We inquired about whether the surgeon has conducted revisional MBS procedures in 2021, 2022, and 2023, and requested data on the volume of these surgeries for each year. To account for variability, we allowed for multiple responses regarding the most common types of revisional/conversional surgeries these surgeons perform (Table 4). Additionally, we correlated these responses with the surgical expertise of the performing surgeon, distinguishing between consultants and specialists.

### Assessing the Utilization of EGD

To better understand the practices related to EGD utilization in asymptomatic patients undergoing bariatric surgery, our survey of collaborating surgeons inquired about their pre- and post-operative use of EGD for these patients (Table 5). Specifically, we sought to determine whether EGD is routinely performed, selectively used, or not used at all, and also delved into the criteria guiding selective use. Factors considered included revisional surgeries, patient characteristics (such as age, sex, or family history of gastrointestinal pathologies), procedural factors (e.g., type of surgeries performed), and any additional reasons influencing EGD use. Other reasons were then explored further by an open-choice question with multiple answers to capture various factors, allowing respondents to freely describe any additional factors influencing their decision to use EGD or a combination of the enlisted factors.

**Table 5:** Survey questions on bariatric surgery practices and response percentages

Question	Answer Choices	Percentage
** Do you routinely offer pre-operative EGD for asymptomatic patients undergoing bariatric surgery? **	Yes, for all patients before bariatric surgery	12.5%
	Yes, on a selective basis	67.5%
	No, I do not routinely offer EGD before bariatric surgery	20%
** On what basis do you selectively offer pre-operative EGD in asymptomatic patients? **	**Patient factors (22.5%)**	
	Age	23.8%
	Sex	11.3%
	Family history	23.8%
	**Procedural factors (3.8%)**	
	LSG	1.3%
	RYGB	6.3%
	OAGB	1.3%
	**Revisional surgery (32.5%)**	
	**Others (8.8%)**	
** Do you routinely offer EGD at 1 year for asymptomatic patients after bariatric surgery? **	Yes, for all patients after bariatric surgery	3.8%
	Yes, on a selective basis	41.3%
	No, I do not routinely offer EGD after bariatric surgery	55%
** On what basis do you selectively offer post-operative EGD in asymptomatic patients at 1 year? **	**Patient factors (10%)**	
	Age	7.5%
	Sex	2.5%
	Family history	7.5%
	**Procedural factors (3.8%)**	
	LSG	3.8%
	RYGB	1.3%
	OAGB	2.5%
	**Revisional surgery (6.3%)**	
	**Others (21.3%)**	
** Do you routinely offer EGD every 2–3 years for patients who have undergone LSG? **	Yes	17.5%
	No	76.3%
	Not Applicable	6.3%
** Do you routinely offer EGD every 2–3 years for patients who have undergone OAGB? **	Yes	20%
	No	65%
	Not Applicable	15%
** Do you routinely offer EGD every 2–3 years for patients who have undergone RYGB? **	Yes	13.8%
	No	72.5%
	Not Applicable	13.8%
** Do you routinely offer EGD every 2–3 years for patients who have undergone SADI-S? **	Yes	3.8%
	No	17.5%
	Not Applicable	78.8%
** Do you routinely offer EGD every 2–3 years for patients who have undergone AGB? **	Yes	6.3%
	No	42.5%
	Not Applicable	51.3%
** Are you aware of the IFSO position statement released in August 2020 on the routine use of EGD in bariatric surgery? **	Yes	61.3%
	No	38.8%

Additionally, our survey aimed to gain insight into the preference for routine EGD in 2–3 years post-operative follow-up for patients who have undergone LSG, OAGB, RYGB, SADI-S, and AGB. The survey questions allowed respondents to indicate whether they routinely use EGD in these post-operative follow-ups, do not use it, or mark it as Not applicable (N/A) in cases where the surgeon had no instances of the specified surgery in the past 2–3 years, or the patients had not reached the 2–3-year follow-up mark yet (Table 5).

### Awareness of the 2020 IFSO Position Statement

The survey also assessed the extent to which MBS surgeons in Egypt are aware of the 2020 IFSO position statement recommendations (Table 5). This evaluation aimed to gauge the level of awareness among surgeons and to compare this knowledge with real-life scenarios and its implementation in the surgical practice in Egypt.

### Data Analysis

Once the responses were disclosed, the data underwent analysis using SPSS version 25. The qualitative data were displayed using percentage and frequency. We utilized the Fisher exact test and the chi-square test to evaluate the relationship between categorical variables. *P*-values less than 0.05 were deemed statistically significant.

## Results

### Baseline Characteristics

An analysis of 80 responses from surgeons at various levels revealed that the majority, 88.8%, were consultants and 11.3% were specialists (Table 1). Among the respondents, 73.8% (*n* = 59) were identified as high caseload experts, indicating that they perform more than 100 cases annually. Additionally, 72.5% of the respondents are members of the ESBS, with 65% reporting past or current membership with IFSO, and 18.8% being current 2024 IFSO members (Table 1).

### The Volume of Bariatric Surgeries Performed Annually Throughout Different Egyptian Institutes

The primary surgeon’s involvement in bariatric surgery has consistently grown over the past few years. In 2021, 46.3% of surgeons reported conducting more than 100 cases, a figure that rose to 52.5% in 2022 and further to 57.5% in 2023. Similarly, the volume of bariatric surgeries performed at respondents’ institutes has also shown a consistent increase, with 57.5% of units conducting more than 100 cases in 2021, 68.8% in 2022, and 73.8% in 2023 (Table 4).

### Trends of Bariatric Procedures Performed

The landscape of primary bariatric surgeries has undergone significant changes in recent years. SG has experienced a steady increase, with over 40% of surgeons performing more than 100 cases in 2023, up from 33.8% in 2021. Primary OAGB procedures exceeding 100 cases also saw a slight uptick, reaching 17.5% in 2023 from 15% in 2021. Conversely, primary RYGB procedures displayed varied trends, with a slight increase in the 25–49 cases category but stable numbers for those performing over 100 cases. However, primary banded procedures such as BSG, BOAGB, and BRYGB maintained low overall numbers and did not show significant growth (Table 4).

In the realm of other MBS performed, the single-anastomosis sleeve ileal (SASI) bypass demonstrated a clear upward trajectory, increasing from 31.3% in 2021 to 35% in 2023, indicating a growing preference among surgeons for this procedure. Conversely, the single-anastomosis sleeve jejunal (SASJ) bypass and plication procedures remained relatively stable over this period. Resectional OAGB and single-anastomosis plication ileal (SAPI) bypass demonstrated a modest but steady rise, while the Adjustable Gastric Band (AGB) procedures saw a slight increase in surgeons performing fewer than 25 cases, but overall frequency remained low (Table 4).

Regarding revisional surgeries, it is evident that revisional RYGB remained the most common procedure, with consistent frequencies ranging from 57.5 to 58.8% over the 3 years. Revisional SG or re-sleeve procedures experienced a minor increase from 2.5% in 2021 to 3.8% in 2022 but declined slightly in 2023. Revisional SASI procedures, on the other hand, saw a notable rise from 1.3% in 2021 to 5% in 2022, maintaining this level into 2023. Other revisional interventions, such as revisional OAGB and revisional BRYGB, showed minimal variation in frequency, indicating a consistent but limited utilization in surgical practice (Table 4).

The surgeons’ expertise level, caseload, and the frequency of revisional bariatric surgeries are strongly correlated. Consultants, who have more experience, are more likely to perform a wider range of revisional surgeries compared to specialists (*P* = 0.008, *P* = 0.003, *P* = 0.041 in the consequent years, respectively) (Table 6). Surgeons with higher caseloads, performing more than 100 cases annually, are significantly more likely to perform complex and higher volumes of revisional procedures (*P* = 0.013, *P* = 0.034, *P* = 0.049 in the consequent years, respectively) (Table 6). This trend has remained consistent over the years, indicating that both the surgeon’s expertise grade (consultant vs. specialist) and caseload are key factors influencing their involvement in revisional surgeries (Table 6).

**Table 6:** Correlation of the Surgeon’s Expertise and surgeons’ caseload with the volume of Revisional Surgeries performed in consecutive years 2021, 2022, and 2023

The volume of Revisional surgeries performed yearly	Surgeon’s Expertise and Certification			Surgeon’s caseload		
	Consultant Surgeon *n *= 71	Specialist Surgeon *n *= 9	Chi-Square (*P*-value)	High caseload Surgeon *n *= 59	Low caseload Surgeon *n *= 21	Chi-Square (*P*-value)
**Revisional Surgeries (2021)**						
< 25 cases	23 (32.4)	4 (44.4)		17 (28.8)	10 (47.6)	
25–49 cases	13 (18.3)	0 (0)	**13.679**	10 (16.9)	3 (14.3)	**12.635**
50–100 cases	16 (22.5)	0 (0)	**(0.008)**	15 (25.4)	1 (4.8)	**(0.013)**
> 100 cases	10 (14.1)	0 (0)		10 (16.9)	0 (0)	
None	9 (12.7)	5 (55.6)		7 (11.9)	7 (33.3)	
**Revisional Surgeries (2022)**						
< 25 cases	24 (33.8)	3 (33.3)		17 (28.8)	10 (47.6)	
25–49 cases	20 (28.2)	0 (0)		17 (28.8)	3 (14.3)	
50–100 cases	9 (12.7)	0 (0)	**15.891**	8 (13.6)	1 (4.8)	**10.423**
> 100 cases	10 (14.1)	0 (0)	**(0.003)**	10 (16.9)	0 (0)	**(0.034)**
None	8 (11.3)	5 (55.6)		7 (11.9)	6 (28.6)	
**Revisional Surgeries (2023)**						
< 25 cases	27 (38)	3 (33.3)		22 (37.3)	8 (38.1)	
25–49 cases	14 (19.7)	0 (0)		11 (18.6)	3 (14.3)	
50–100 cases	9 (12.7)	0 (0)	**9.973**	8 (13.6)	1 (4.8)	**9.547**
> 100 cases	11 (15.5)	0 (0)	**(0.041)**	11 (18.6)	0 (0)	**(0.049)**
None	10 (14.1)	4 (44.4)		7 (11.9)	7 (33.3)	
**Are you aware of the IFSO position statement released in August 2020 on the routine use of EGD in bariatric surgery?**						
Yes	43 (60.6)	6 (66.7)	0.125	36 (61)	13 (61.9)	0.005
No	28 (39.4)	3 (33.3)	(1.000)	23 (39)	8 (38.1)	(1.000)

### Pre-operative Use of EGD

**Fig. 2:**
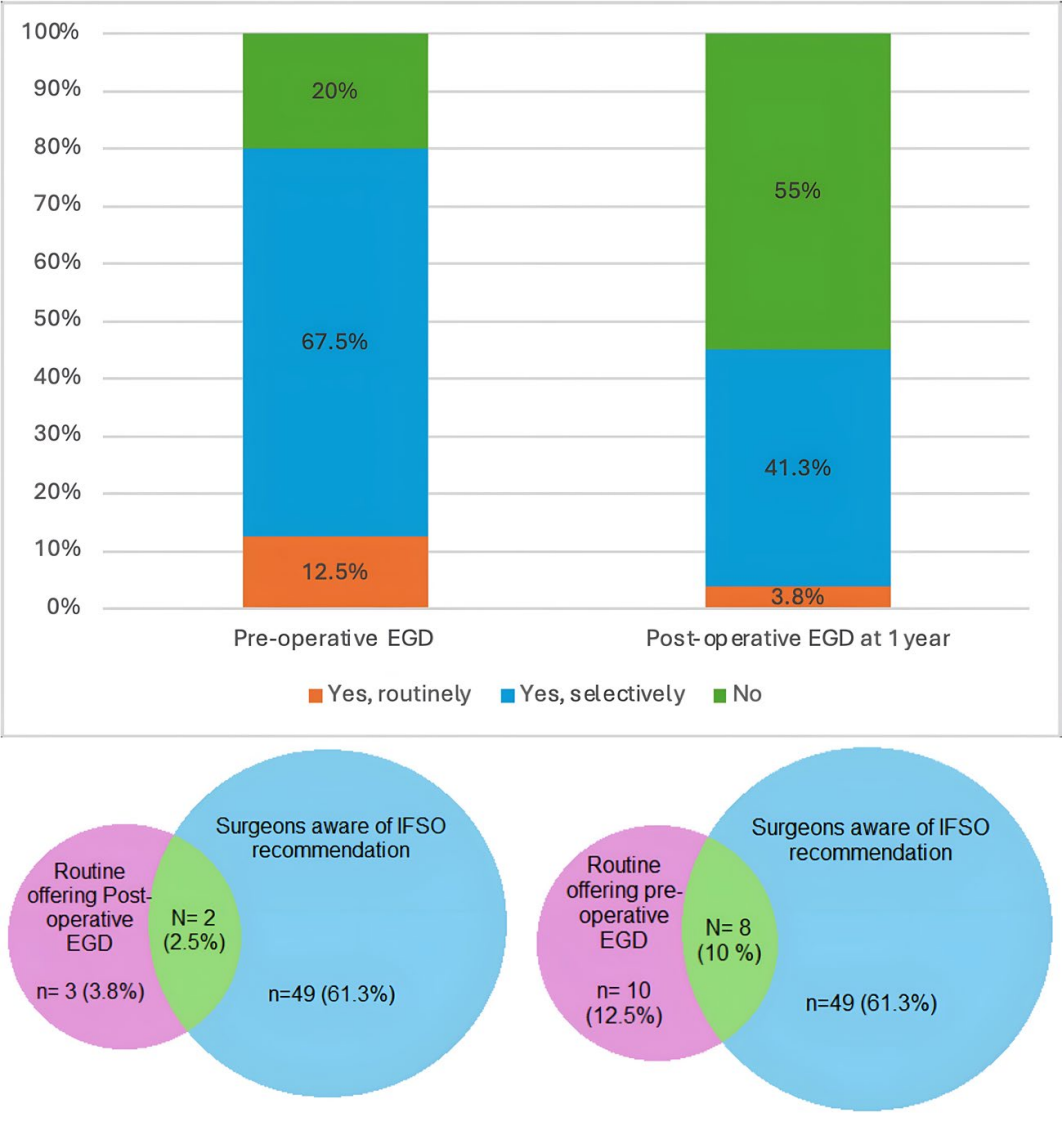
(**a**) A stacked bar chart showing the Bariatric surgeons’ practice on the utilization of pre-and post-operative EGD at 1 year by percentages. (**b**) Two Venn diagrams showing the proportion of surgeons routinely offering post-operative EGD (the left Venn diagram) and pre-operative EGD (the right Venn diagram) and who are aware of IFSO recommendations.

The utilization of EGD before bariatric surgery among surgeons for asymptomatic patients showed wide variation (Table 5). A majority of respondents (67.5%) reported selective use of EGD based on specific criteria including revisional surgeries, patient factors (age, sex, family history), and procedural factors (type of bariatric surgery such as LSG, RYGB, or OAGB). Only 12.5% of surgeons routinely conducted EGD for all patients before bariatric surgery, while 20% did not offer it routinely (Fig. 2). The decision to selectively perform EGD was primarily influenced by the type of surgery planned, with revisional surgeries being a major determinant (32.5%). Other commonly cited factors included family history of gastrointestinal disease (23.8%) and patient age (22.5%) (Table 5).

### Post-operative Use of EGD

In terms of the utilization of post-operative EGD, our survey revealed that 55% of surgeons do not routinely offer EGD after one year for asymptomatic patients. Additionally, 41.3% offer it selectively based on patient-specific criteria, while only 3.8% routinely offer EGD for all patients at the 1-year mark (Fig. 2). Selective criteria for post-operative EGD include revisional surgeries (6.3%), increased patient age (7.5%), and any abnormal findings in the initial pre-operative EGD (21.3%) (Table 5).

In the realm of the routine use of EGD every 2–3 years for different bariatric procedures, the majority of respondents do not routinely perform EGD for procedures like SG (76.3%), OAGB (65%), or RYGB (72.5%). Only a small percentage of surgeons continue to offer routine EGD for patients who underwent procedures such as SADI-S (3.8%) and AGB (6.3%) (Table 5).

### The Institutional Types and Volume’s Influence on the Utilization of EGD

**Fig. 3:**
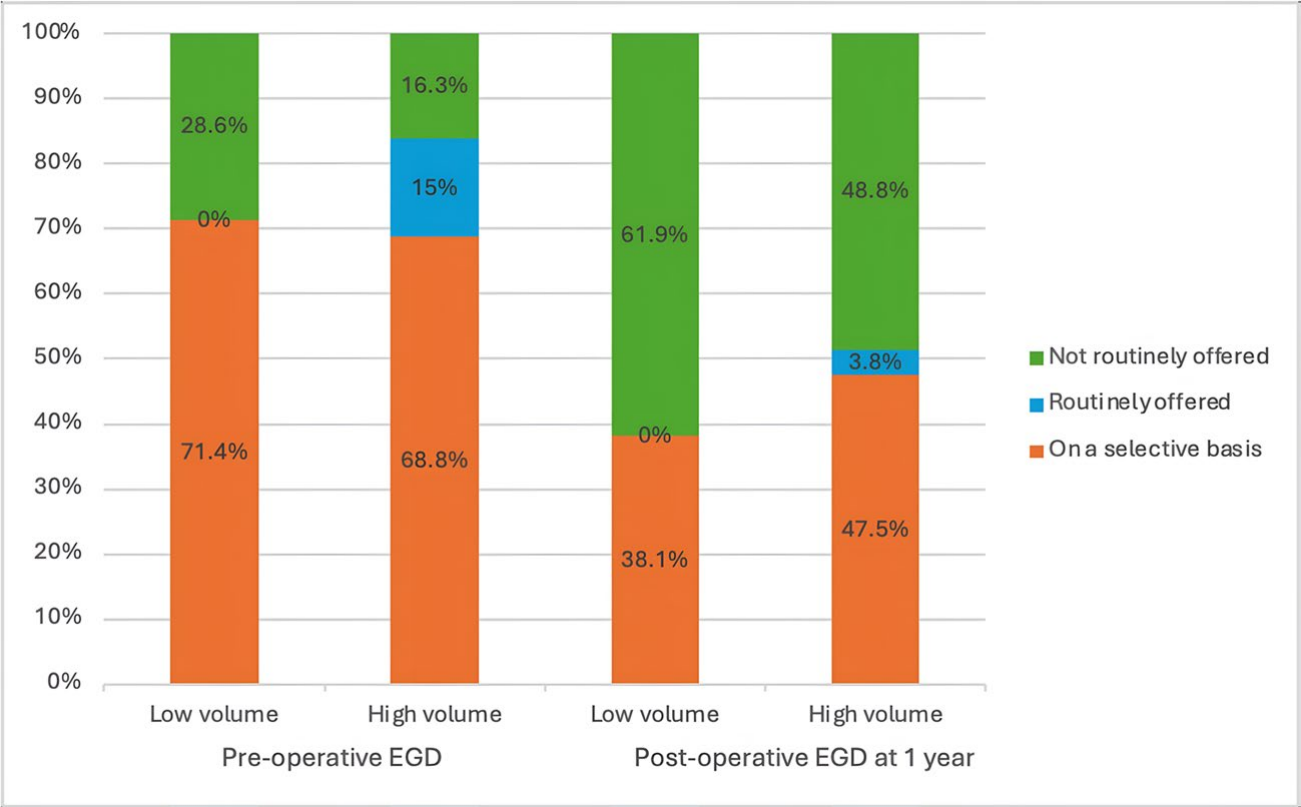
A stacked bar chart showing the relationship between the volume of bariatric surgery cases per institution and individual surge on’s practice on routine pre-operative EGD and 1-year post-operative EGD

**Fig. 4:**
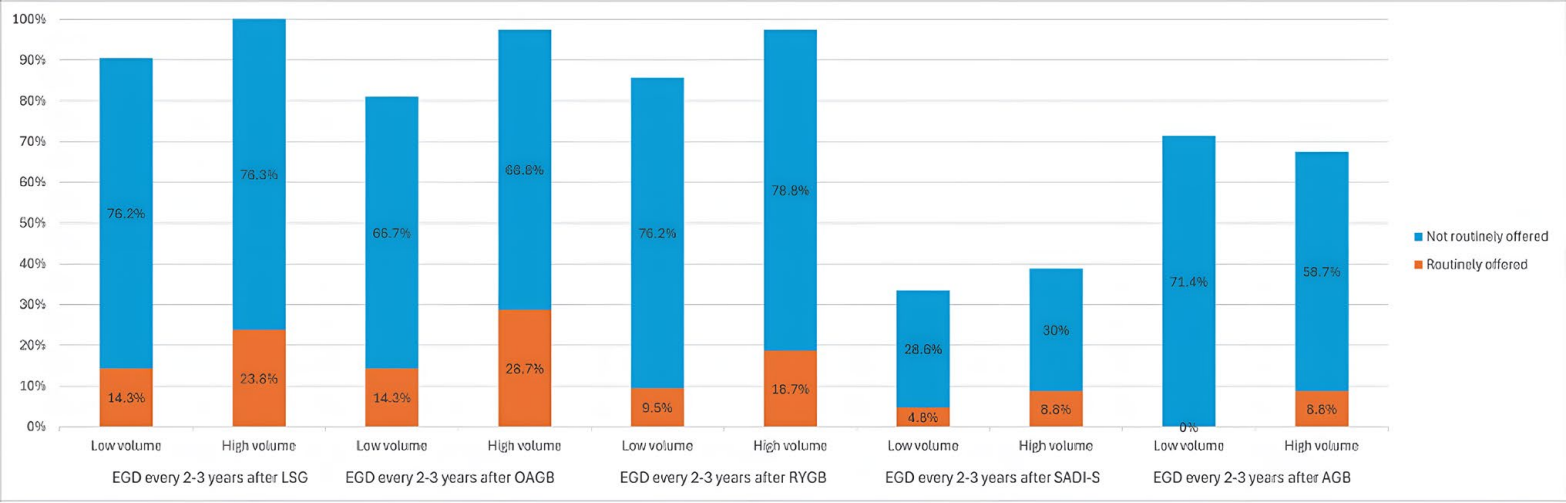
A stacked bar chart showing the relationship between the volume of bariatric surgery cases per institution and individual surgeon’s practice on routine surveillance 2–3 years post-operative EGD to different MBS procedures

The chi-square test analysis did not show a statistically significant association between the annual volume of bariatric cases per institution and the regular offering of pre-and post-operative EGD or surveillance EGD for different procedures (Table 7). However, there was an observed trend indicating that surgeons in higher-volume centers were more likely to selectively offer pre-operative EGD compared to those in lower-volume centers at preoperative EGD, 1-year postoperative EGD (Fig. 3), and 2–3 years surveillance EGD (Fig. 4).

**Table 7:** Chi-square test showing no significant correlation between the volume of the centers, and the utilization of EGD in the pre-and post-operative settings

Questions related to the utilization of EGD in both pre-and post-operative settings	High Volume Center *n *= 80	Low Volume Center *n *= 21	Chi-Square	*P*-value
**Do you routinely offer pre-operative EGD for asymptomatic patients undergoing bariatric surgery?**				
- Yes, on a selective basis.	55 (68.8)	15 (71.4)	4.510	0.105
- Yes, for all patients before bariatric surgery.	12 (15)	0 (0)		
- No, I do not routinely offer EGD before bariatric surgery.	13 (16.3)	6 (28.6)		
**Do you routinely offer EGD at 1 year for asymptomatic patients after bariatric surgery?**				
- Yes, on a selective basis.	38 (47.5)	8 (38.1)	1.670	0.434
- Yes, for all patients after bariatric surgery at 1 year.	3 (3.8)	0 (0)		
- No, I do not routinely offer EGD after bariatric surgery for 1 year.	39 (48.8)	13 (61.9)		
**Do you routinely offer EGD every 2–3 years for patients who have undergone LSG?**				
Yes	19 (23.8)	3 (14.3)	0.563	0.553
No	61 (76.3)	16 (76.2)		
**Do you routinely offer EGD every 2–3 years for patients who have undergone OAGB?**				
Yes	23 (28.7)	3 (14.3)	0.984	0.385
No	55 (68.8)	14 (66.7)		
**Do you routinely offer EGD every 2–3 years for patients who have undergone RYGB?**				
Yes	15 (18.7)	2 (9.5)	0.662	0.515
No	63 (78.8)	16 (76.2)		
**Do you routinely offer EGD every 2–3 years for patients who have undergone SADI-S?**				
Yes	7 (8.8)	1 (4.8)	0.236	1.000
No	24 (30)	6 (28.6)		
**Do you routinely offer EGD every 2–3 years for patients who have undergone AGB?**				
Yes	7 (8.8)	0 (0)	2.164	0.333
No	47 (58.7)	15 (71.4)

**Fig. 5:**
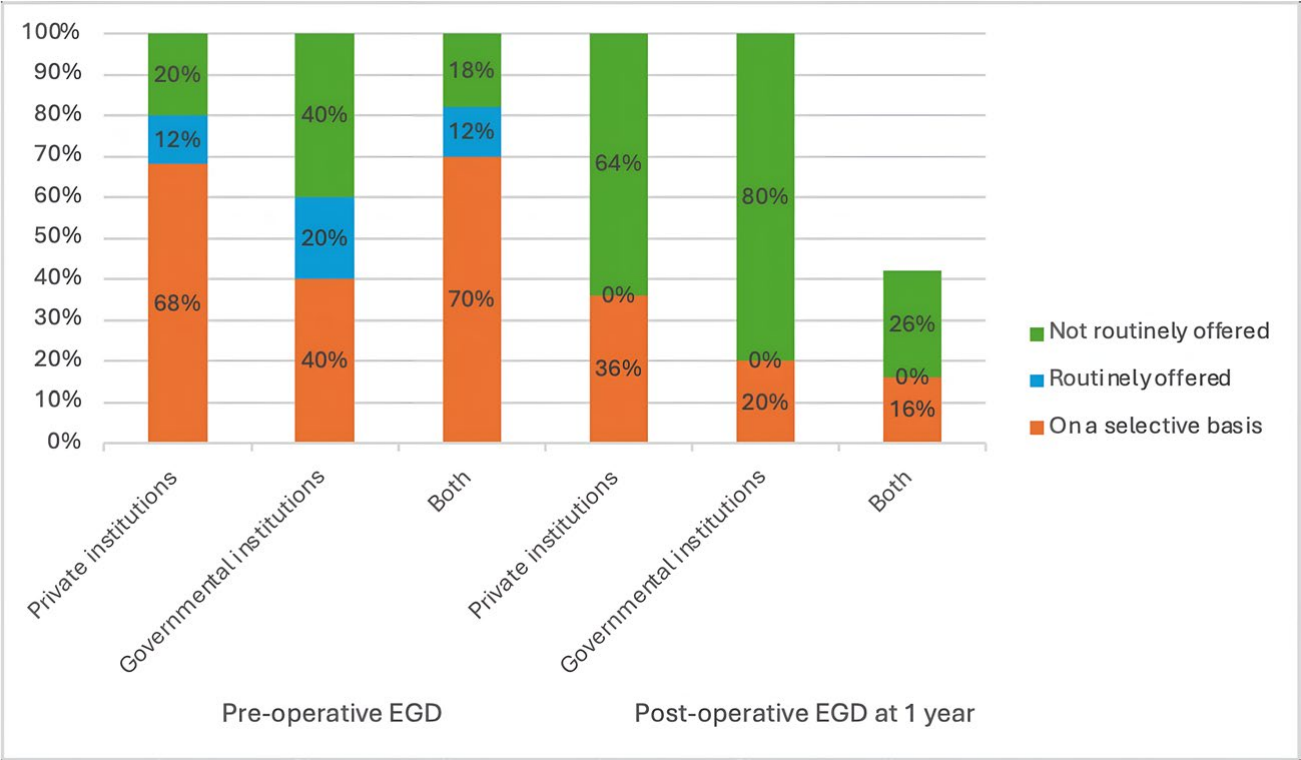
A stacked bar chart showing the relationship between the types of institutions (governmental institute, private institute, and combined institutional settings) and individual surgeon’s practice on routine pre-operative EGD and 1-year post-operative EGD

Additionally, there were no significant differences in EGD practices between private, and governmental institutions, and those performing in both types of institutions (Table 8). Nevertheless, 68% of surgeons in private institutions and 70% in combined settings offered pre-operative EGD on a selective basis, compared to only 40% in governmental institutions (Fig. 5). Conversely, a higher percentage of surgeons in governmental institutions (20%) offer routine pre-operative EGD compared to those in private institutes (12%), and combined institutional settings (12%) (Fig. 5).

**Table 8:** Chi-square test showing no significant correlation between the types of institutions, and the utilization of EGD in the pre-and post-operative settings

Questions related to the utilization of EGD in both pre-and post-operative settings	Private institutions *n *= 25	Governmental institutions *n *= 5	Both Private and Governmental *n *= 50	Chi-Square	*P*-value
**Do you routinely offer pre-operative EGD for asymptomatic patients undergoing bariatric surgery?**					
- Yes, on a selective basis.	17 (68)	2 (40)	35 (70)	1.947	0.745
- Yes, for all patients before bariatric surgery.	3 (12)	1 (20)	6 (12)		
- No, I do not routinely offer EGD before bariatric surgery.	5 (20)	2 (40)	9 (18)		
**Do you routinely offer EGD at 1 year for asymptomatic patients after bariatric surgery?**					
- Yes, on a selective basis.	9 (36)	1 (20)	8 (16)	4.170	0.384
- Yes, for all patients after bariatric surgery at 1 year.	0 (0)	0 (0)	0 (0)		
- No, I do not routinely offer EGD after bariatric surgery for 1 year.	16 (64)	4 (80)	13 (26)		
**Do you routinely offer EGD every 2–3 years for patients who have undergone LSG?**					
Yes	4 (16)	1 (20)	39 (78)	0.009	0.995
No	18 (72)	4 (80)	9 (18)		
**Do you routinely offer EGD every 2–3 years for patients who have undergone OAGB?**					
Yes	5 (20)	0 (0)	11 (22)	0.977	0.614
No	16 (64)	3 (60)	33 (66)		
**Do you routinely offer EGD every 2–3 years for patients who have undergone RYGB?**					
Yes	2 (8)	0 (0)	9 (18)	2.008	0.366
No	20 (80)	3 (60)	35 (70)		
**Do you routinely offer EGD every 2–3 years for patients who have undergone SADI-S?**					
Yes	1 (4)	0 (0)	2 (4)	0.195	1.000
No	3 (12)	0 (0)	11 (22)		
**Do you routinely offer EGD every 2–3 years for patients who have undergone AGB?**					
Yes	3 (12)	0 (0)	2 (4)	1.950	0.377
No	10 (40)	2 (40)	22 (44)

**Fig. 6:**
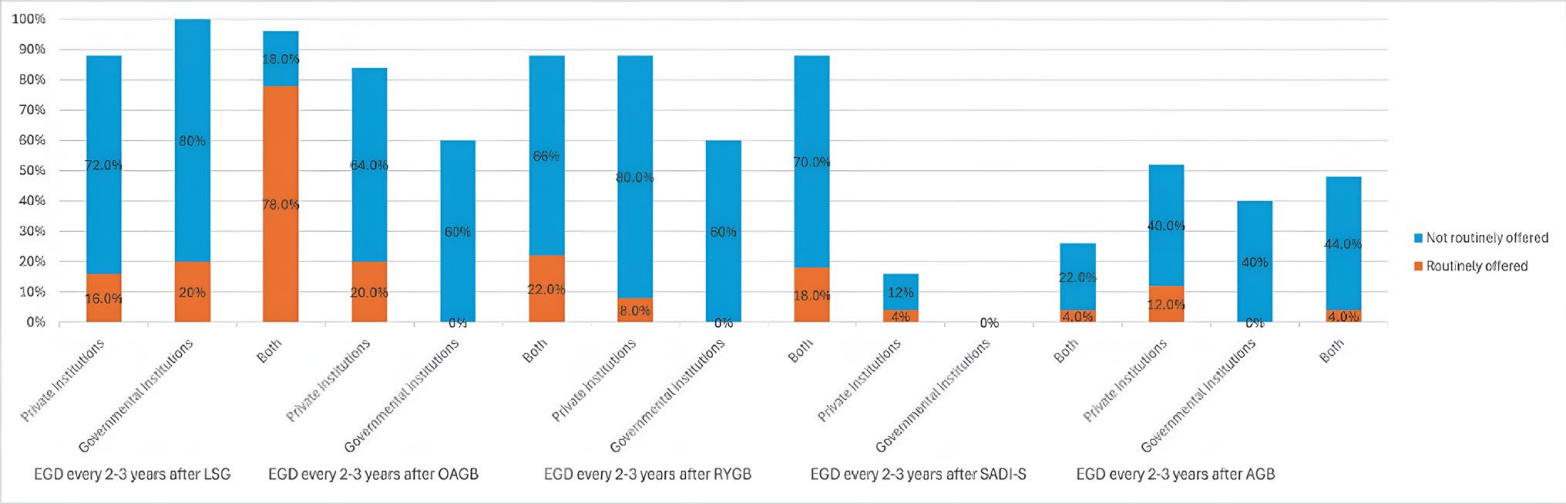
A stacked bar chart showing the relationship between the types of institutions (governmental institute, private institute, and combined institutional settings) and individual surgeon’s practice on routine surveillance 2–3 years post-operative EGD to different MBS procedures

Surveillance EGD offered 2–3 years after SG, OAGB, and RYGB in the combined institutional settings were higher than those offered in private and governmental institutes (Fig. 6). However, surveillance EGD was offered less in private institutes after SG than in governmental institutes but was offered more after OAGB and RYGB than in governmental institutes (Fig. 6). These findings suggest that while the type of institution does not significantly impact EGD practices overall, there are variations in how frequently EGD is offered, with private and combined institutions favoring selective use more than governmental ones. Additionally, governmental institutes tend to offer more routine preoperative EGD with no routine follow-up EGD either at 1-year follow-up or at 2–3 years follow-up.

### Awareness of the 2020 IFSO Position Statement Recommendations and Its Impact

The IFSO 2020 recommendations on the routine use of EGD in bariatric surgery seem to have had little influence on the actual implementation of EGD both pre- and post-operatively among surgeons in Egypt (Fig. 2). Among surgeons who are aware of the recommendations, 61.2% offer EGD selectively before bariatric surgery, compared to 77.4% of those who are unaware of the recommendations, indicating no significant correlation between recommendations awareness and pre-operative EGD practices (chi-square = 2.598, *P* = 0.273) (Table 9).

**Table 9:** Chi-square test showing no significant correlation between the Surgeon’s awareness of the IFSO recommendations, and the utilization of EGD in the pre-and post-operative settings

Questions related to the utilization of EGD in both pre-and post-operative settings	Aware of the IFSO Recommendations *n *= 49	Not aware of the IFSO recommendations *n *= 31	Chi-Square	*P*-value
**Do you routinely offer pre-operative EGD for asymptomatic patients undergoing bariatric surgery?**				
- Yes, on a selective basis.	30 (61.2)	24 (77.4)	2.598	0.273
- Yes, for all patients before bariatric surgery.	8 (16.3)	2 (6.5)		
- No, I do not routinely offer EGD before bariatric surgery.	11 (22.4)	5 (16.1)		
**Do you routinely offer EGD at 1 year for asymptomatic patients after bariatric surgery?**				
- Yes, on a selective basis.	21 (42.9)	12 (38.7)	0.203	0.904
- Yes, for all patients after bariatric surgery at 1 year.	2 (4.1)	1 (3.2)		
- No, I do not routinely offer EGD after bariatric surgery for 1 year.	26 (53.1)	18 (58.1)		
**Do you routinely offer EGD every 2–3 years for patients who have undergone LSG?**				
Yes	9 (18.4)	5 (16.1)	0.063	1.000
No	37 (75.5)	24 (77.4)		
**Do you routinely offer EGD every 2–3 years for patients who have undergone OAGB?**				
Yes	12 (24.5)	4 (12.9)	1.246	0.377
No	31 (63.3)	21 (67.7)		
**Do you routinely offer EGD every 2–3 years for patients who have undergone RYGB?**				
Yes	6 (12.2)	5 (16.1)	0.482	0.511
No	38 (77.6)	20 (80)		
**Do you routinely offer EGD every 2–3 years for patients who have undergone SADI-S?**				
Yes	1 (2.04)	2 (6.5)	0.977	0.537
No	9 (18.4)	5 (16.1)		
**Do you routinely offer EGD every 2–3 years for patients who have undergone AGB?**				
Yes	4 (8.2)	1 (3.2)	1.298	0.363
No	18 (36.7)	16 (51.6)		

Similarly, for post-operative EGD at 1 year, 42.9% of aware surgeons offer it selectively, while 38.7% of unaware surgeons do so, reflecting a minimal difference (chi-square = 0.203, *P* = 0.904). Furthermore, in terms of the frequency of offering EGD every 2–3 years for patients with specific bariatric procedures (e.g., SG, OAGB, RYGB), no significant differences were observed between those aware and unaware of the guidelines, as indicated by high *P*-values (Table 9). This indicates that awareness of the IFSO recommendations does not substantially influence the clinical decisions regarding the routine use of EGD in bariatric surgery practices in Egypt.

## Discussion

Our survey represents Egypt’s first national effort to examine EGD use in MBS and the challenges MBS surgeons face in adhering to optimal healthcare recommendations. It provides insights into current trends in MBS and surgeon knowledge of recent recommendations.

Since its founding, IFSO has played a pivotal role in advancing MBS worldwide by promoting best practices, facilitating knowledge exchange, and fostering professional development. This has significantly contributed to the standardization and improvement of MBS techniques, training, and outcomes across various regions, including Egypt [11].

With the establishment of the ESBS as a member Society of the IFSO in 1999, the field of MBS has experienced substantial growth in Egypt [12]. This growth has led to an increased number of gastrointestinal surgeons specializing in MBS under the auspices of the ESBS. The rising prevalence of MBS procedures and surgeons, as well as their positive impact on patients, has further boosted the demand for MBS, thereby attracting more practitioners to the field. However, this surge has introduced concerns regarding potential variations in the quality of care, with some young surgeons performing MBS procedures without affiliating with the local national society or adhering to IFSO guidelines. Membership in professional societies like ESBS and IFSO is typically tied to adherence to established clinical guidelines and best practices, ensuring that all surgeons meet minimum quality standards and follow globally recognized guidelines [13–16].

Despite the high expertise level of bariatric surgeons in our survey with 88.8% of respondents being consultants and 73.8% performing more than 100 surgeries annually, the findings indicate that high surgical volumes alone do not guarantee uniform adherence to standardized practices. However, the data suggests that surgeons with higher surgical volumes tend to adhere more to IFSO recommendations compared to those with lower volumes, likely due to greater exposure to varied clinical scenarios and the need to stay updated with evolving standards of care [5]. This underscores the importance of continuous professional development, including active participation in professional societies such as ESBS and IFSO, and participation in specialized training and observership programs [17, 18].

Structured fellowship training and adherence to professional guidelines are vital in improving the quality of bariatric surgery [17, 18]. A recent International Experts’ Consensus suggests that completing a dedicated fellowship and conducting a specific number of procedures, such as 25–50 for SG, 50–75 for OAGB, and 75–100 for RYGB, are crucial for gaining proficiency [18]. Additionally, Continuous education, certification, and active participation in professional organizations are necessary to uphold high standards and enhance patient outcomes [17]. Furthermore, Systematic recording of patient outcomes and involvement in national or global registries are recommended for identifying areas that need improvement and for mentoring less experienced surgeons [17]. Thus, aligning local practices with these global standards can minimize disparities in care and improve overall outcomes, emphasizing the importance of establishing structured training programs and mandatory memberships in Egypt to uphold international guidelines.

Our study highlights the increasing demand for bariatric procedures in Egypt, along with the challenges in implementing routine EGD due to economic constraints. These findings align with international reports where economic barriers often determine adherence to recommended guidelines [19]. The limited use of EGD in developing countries is often linked to out-of-pocket healthcare payments and institutional funding restrictions [19, 20]. Addressing these challenges requires structured training, greater financial support for diagnostic tools, and improved guideline dissemination tailored to economic realities [7, 17, 18, 20, 21].

Our data indicates that 68% of surgeons in private institutions and 70% of surgeons in both private and governmental institutions in Egypt incorporate selective pre-operative EGD, compared to only 40% of surgeons in governmental institutions. A slightly larger percentage of routine pre-operative EGD offered in governmental institutions (20%) than those in both the private sector (12%) and the combined institutional settings (12%) (Fig. 5). However, Routine post-operative EGD was not offered in governmental institutions either at 1-year follow-up (Fig. 5) or at 2–3 years follow-up (Fig. 6). This disparity suggests that financial limitations in governmental institutions may restrict the routine use of EGD, which could be seen as an additional cost burden in an already expensive surgical procedure.

Our findings reveal that awareness of these recommendations does not always equate to consistent application. Although most respondents were familiar with IFSO recommendations as members of IFSO or ESBS, this awareness did not significantly correlate with the actual use of EGD. This indicates that knowledge of international guidelines alone is insufficient to change practice patterns, especially when economic constraints are a significant barrier [22–24].

These findings also highlight the financial implications of including EGD expenses on top of the already costly bariatric surgery, especially in government healthcare settings with constrained budgets and resources [25]. In these facilities, preoperative EGD was provided in 60% of cases (20% routinely and 40% on a case-by-case basis), while postoperative surveillance EGD did not exceed 20% collectively either selectively (20%) or as a routine procedure (0%).

Conversely, the practice of EGD in private and combined institutional settings tends to favor the selective usage of EGD in both preoperative and postoperative settings. Although private institutions may have more flexibility in resource allocation based on individualized patient financial capacity, the utilization of EGD remains restricted due to financial considerations. This underscores the predominant influence of economic factors over clinical guidelines in this context [26].

Additionally, the high prevalence of obesity in Egypt exacerbates these economic challenges. According to a survey conducted under the “100 Million Health” initiative, approximately 39.8% of adult Egyptians are classified as obese, with a higher prevalence in females (49.5%) compared to males [27–29]. This high obesity rate increases the demand for MBS, which in turn puts pressure on the healthcare system to provide cost-effective treatments [27, 28, 30]. Consequently, many surgeons choose to use EGD selectively, primarily in cases where there is a suspicion of complications or incidental pathologies, rather than as a routine diagnostic tool for all patients​ [31, 32].

This selective use reflects a broader trend where financial considerations often outweigh clinical guidelines, even when routine use could potentially improve patient outcomes [6]. Consequently, efforts to improve adherence to best practices must also address these financial limitations, potentially through policy changes or funding initiatives that make essential diagnostic procedures like EGD more accessible.

At the 19th annual conference of the ESBS, the president of the congress, Professor Khaled Gawdat [33], highlighted that the need for a comprehensive national Egyptian Bariatric Surgery Registry is important and challenging [26]. There is currently no national registry to accurately capture the prevalence of obesity or the number of surgeries performed, making it difficult to evaluate trends and outcomes objectively [7, 34, 35]. Available data only provide rough estimates of an increasing trend in both obesity and the number of surgeries, which impairs the development of effective public health strategies and interventions [7, 34]. IFSO has also highlighted severe limitations in both national and international survey registration, emphasizing the urgent need for a national registry in Egypt [7, 34]. Such a registry would provide accurate, evidence-based insights into the true extent of bariatric surgeries and their outcomes, facilitating better policy-making, resource allocation, and clinical management [21].

Thus the decision-making process around the use of EGD is influenced by both the economic factors and the absence of comprehensive data. The survey indicates that 67.5% of surgeons use EGD selectively, based on specific criteria such as patient age, family history, and the type of surgery planned. Only a small percentage of surgeons routinely use EGD for all patients, largely due to concerns over costs and the perceived lack of necessity in asymptomatic patients. The lack of specific data on the prevalence of incidental pathologies in Egypt complicates these decisions further, as surgeons must balance the risk of missing significant findings against the financial burden of routine testing. This underscores the critical need for more data to inform clinical practices effectively.

While the overall trends in bariatric surgery in Egypt generally mirror global patterns [7, 35], certain differences are notable, particularly in the types of procedures gaining popularity. For example, the LSG is currently the most commonly performed bariatric surgery in Egypt, reflecting a trend observed worldwide [7, 35]. LSG has become the preferred choice for many surgeons due to its relatively straightforward technique, lower complication rates, and substantial evidence supporting its efficacy in achieving significant weight loss and improving obesity-related comorbidities [36].

Furthermore, “Sleeve Plus” surgeries, such as the single-anastomosis sleeve ileal (SASI) bipartition, have also gained popularity, driven partly by aggressive marketing campaigns that highlight its perceived effectiveness [37–41]. Despite this, there are conflicting views on the long-term outcomes of the SASI procedure in Egypt; it remains relatively understudied, and some reports suggest potential risks, including reflux and malnutrition [42–44]. In contrast, other types of metabolic and bariatric surgeries (MBS), like the single-anastomosis sleeve jejunal (SAS-J) bypass, are performed less frequently, reflecting a cautious approach due to limited data on their long-term outcomes and safety profiles [45, 46]. This increased number of other MBS procedures shown in our survey would increase concerns about the ethical considerations adopted for experimental surgeries in Egypt, which are still under study, and are yet to be determined as to whether become profoundly adopted surgical techniques or will be obsolete and mentioned historically in literature [21, 47].

Meanwhile, there is a growing trend in revisional surgeries, with a strong correlation between surgeon expertise and the frequency of these procedures. Revisional bariatric surgeries are increasingly performed in Egypt to address complications, inadequate weight loss, or weight regain following the initial surgery [48, 49]. Data suggests that experienced surgeons are more likely to handle these complex secondary operations, which require advanced skills and a comprehensive understanding of both the primary and revision procedures [48, 49]. Studies have shown that surgeons with higher case volumes and specialized training are better equipped to manage the technical challenges and potential complications associated with revisional surgeries, aligning with existing literature that suggests more experienced surgeons tend to perform these operations more frequently [48–50]. Our data coincided with the literature, where more expert surgeons are undergoing revisional surgeries than those with lower expertise.

We must recognize the limitations of our study. The response rate of 53.3% is relatively low, potentially introducing bias into the results. Although efforts to minimize selection bias were made by including non-members of the ESBS, the limited number of respondents still reduces the generalizability of our findings, yet the variability of responses from different centers across Egypt mitigates this limitation. Additionally, our data was reliant on self-reported information, which may have been influenced by recall bias or inaccuracies. The survey predominantly included consultants (88.8%) with only 11.3% being specialists, indicating differing levels of expertise that could have influenced reported practices and affected the general applicability of the results. The absence of a comprehensive national data registry presents a significant challenge in drawing broader conclusions from the collected data. These limitations underscore the need for more extensive and representative research to paint a clearer picture of bariatric surgery practices in Egypt.

To enhance the quality and consistency of bariatric surgery in Egypt, future research efforts should center around gaining a more comprehensive understanding of economic barriers, establishing a national registry to capture accurate surgical trend data, and consistently reporting incidental findings. Moreover, there should be initiatives to integrate IFSO registry practices in Egypt, ensuring the approval of specific bariatric surgeries by recognized societies to uphold high standards. Additionally, there should be the formation of observership programs and continuing training programs designed by the ESBS and the IFSO, aiming to both train young surgeons and implement good practice among working surgeons.

## Conclusion

The increasing volume of bariatric procedures in Egypt reflects the rising demand for obesity management; however, there are notable deficiencies in the utilization of critical diagnostic modalities such as EGD. Our study reveals that the application of EGD in the context of bariatric surgery in Egypt does not adhere fully to the IFSO recommendations, likely due to economic barriers. To enhance patient outcomes and align local practices with international benchmarks, it is essential to implement strategies that specifically target these financial and informational deficiencies.

Establishing a national registry is a pivotal step toward promoting consistency and elevating the quality of bariatric care across Egypt. Additionally, addressing economic constraints is vital for improving access to necessary diagnostic tools. The introduction of a certified bariatric training curriculum, coupled with enhanced observership programs, will facilitate the adoption of contemporary guidelines in surgical practice in Egypt. These initiatives are crucial to ensuring that all patients benefit from optimal care informed by accurate data and consistent with global best practices.

## Supplementary Information

Below is the link to the electronic supplementary material.

Supplementary file1 (DOCX 51 KB)

## Data Availability

All data generated in this article is available in the article and in Supplementary File 2.

## Abbreviations

AGB: Adjustable gastric band
BSG: Banded sleeve gastrectomy
BRYGB: Banded Roux-en-Y gastric bypass
BOAGB: Banded one-anastomosis gastric bypass
EGD: Esophagogastroduodenoscopy
ESBS: Egyptian Society of Bariatric Surgeons
IFSO: International Federation for the Surgery of Obesity and Metabolic Disorders
MBS: Metabolic and bariatric surgery
OAGB: One-anastomosis gastric bypass
RYGB: Roux-en-Y gastric bypass
SADI-S: Single-anastomosis duodeno–ileal bypass with sleeve gastrectomy
SAPI: Single-anastomosis plication with ileal bypass
SASJ: Single-anastomosis sleeve jejunal bypass
SASI: Single-anastomosis sleeve ileal bypass/bipartition
SG: Sleeve gastrectomy
