# Supplementary material for: Trends and Practices in Bariatric Surgery in Egypt: Insights on Esophagogastroduodenoscopy (EGD) Utilization and Surgical Volumes
Source: Obes Surg. 2025 May 20;35(7):2528–48. doi: 10.1007/s11695-025-07846-0 (PMC12271285; doi:10.1007/s11695-025-07846-0)
Supplement: Supplementary file 1 — Supplementary file1 (DOCX 51 KB) [file 11695_2025_7846_MOESM1_ESM.docx]

**Figure S1:** Distribution of Professional Grades Among Bariatric Surgeons in Egypt

**Figure S2:** The volume of bariatric surgery the respondent is involved in each year as a primary surgeon

**Figure S3:** The volume of bariatric surgery undertaken at the surgeon’s center

**Figure S3:** Trends of Bariatric Procedures Performed in 2021

**Figure S4:** Trends of Bariatric Procedures Performed in 2022

**Figure S5:** Trends of Bariatric Procedures Performed in 2023

| **Table** | **N(%)** |
| --- | --- |
| **Do you routinely offer pre-operative EGD for asymptomatic patients undergoing bariatric surgery?**  Yes, on a selective basis.  Yes, for all patients before bariatric surgery.  No, I do not routinely offer EGD before bariatric surgery. | 54 (67.5)  10 (12.5)  16 (20) |
| **On what basis do you selectively offer pre-operative EGD in asymptomatic patients?**  - Revisional surgery  - Patient factors *  Age  Sex  Family history  - Procedural factors *  LSG  RYGB  OAGB  - Others *  Revisional Surgery  Family history  Procedural Factors  increased Age  Routine EGD for University Hospital patients | 26 (32.5)  18 (22.5)  19 (23.8)  9 (11.3)  19 (23.8)  3 (3.8)  1 (1.3)  5 (6.3)  1 (1.3)  7 (8.8)  7 (8.8)  5 (6.3)  2 (2.5)  4 (5)  1 (1.3) |
| **Do you routinely offer EGD at 1 year for asymptomatic patients after bariatric surgery?**  Yes, on a selective basis.  Yes, for all patients after bariatric surgery at 1 year.  No, I do not routinely offer EGD after bariatric surgery for 1 year. | 33 (41.3)  3 (3.8)  44 (55) |
| **On what basis do you selectively offer post-operative EGD in asymptomatic patients at 1 year?**  - Revisional surgery  - Patient factors *  Age  Sex  Family history  - Procedural factors *  LSG  RYGB  OAGB  - Others *  -Symptomatic patients  -Symptomatic patients, For follow-up of complicated patients  -Symptomatic patients, pre-operative abnormalities on EGD, for patients involved in research protocols  -Patient Factors and Procedural Factors  -Revisional cases, symptomatic Patients | 5 (6.3)  8 (10)  6 (7.5)  2 (2.5)  6 (7.5)  3 (3.8)  3 (3.8)  1 (1.3)  2 (2.5)  17 (21.3)  12 (15)  2 (2.5)  1 (1.3)  1 (1.3)  1 (1.3) |

**Table S1:** Patterns of EGD Utilization Among Bariatric Surgeons

| **Table** | **N (%)** |
| --- | --- |
| **Do you routinely offer EGD every 2–3 years for patients who have undergone LSG?**  Yes  No  N/A | 14 (17.5)  61 (76.3)  5 (6.3) |
| **Do you routinely offer EGD every 2–3 years for patients who have undergone OAGB?**  Yes  No  N/A | 16 (20)  52 (65)  12 (15) |
| **Do you routinely offer EGD every 2–3 years for patients who have undergone RYGB?**  Yes  No  N/A | 11 (13.8)  58 (72.5)  11 (13.8) |
| **Do you routinely offer EGD every 2–3 years for patients who have undergone SADI-S?**  Yes  No  N/A | 3 (3.8)  14 (17.5)  63 (78.8) |
| **Do you routinely offer EGD every 2–3 years for patients who have undergone AGB?**  Yes  No  N/A | 5 (6.3)  34 (42.5)  41 (51.3) |
| **Are you aware of the IFSO position statement released in August 2020 on the routine use of EGD in bariatric surgery?**  Yes  No | 49 (61.3)  31 (38.8) |

**Table S2:** Routine EGD Use Every 2-3 Years After Different Bariatric Procedures

**Figure S6:** Frequency of EGD Every 2-3 Years Following Different Bariatric Procedures
